# RETRACTION: Effects of Clinical Nursing Pathway on Surgical Site Wound Infection in Patients Undergoing Acute Appendicitis Surgery: A Meta‐Analysis

**DOI:** 10.1111/iwj.70281

**Published:** 2025-03-03

## Abstract

**RETRACTION:** ZhangQ. , ZhangL.‐H. , WuJ.‐L. , and YangF.‐Y. , “Effects of Clinical Nursing Pathway on Surgical Site Wound Infection in Patients Undergoing Acute Appendicitis Surgery: A Meta‐Analysis,” International Wound Journal 21, no. 4 (2024): e14600, 10.1111/iwj.14600.38146201 PMC10961861

The above article, published online on 25 December 2023, in Wiley Online Library (http://onlinelibrary.wiley.com/), has been retracted by agreement between the journal Editor in Chief, Professor Keith Harding; and John Wiley & Sons Ltd. A third party reported to the publisher that several references included in the article were unavailable. An investigation by the publisher verified that some references did not have available online records. Following additional investigation, all parties have concluded that this article was accepted solely on the basis of a compromised peer review process. In view of the clear evidence of compromised peer review, the parties agreed that the paper must be retracted. The authors did not respond to our notice regarding the retraction.

**RETRACTION:** Q. Zhang , L.‐H. Zhang , J.‐L. Wu , and F.‐Y. Yang , “Effects of Clinical Nursing Pathway on Surgical Site Wound Infection in Patients Undergoing Acute Appendicitis Surgery: A Meta‐Analysis,” International Wound Journal 21, no. 4 (2024): e14600, 10.1111/iwj.14600.38146201 PMC10961861

The above article, published online on 25 December 2023, in Wiley Online Library (http://onlinelibrary.wiley.com/), has been retracted by agreement between the journal Editor in Chief, Professor Keith Harding; and John Wiley & Sons Ltd. A third party reported to the publisher that several references included in the article were unavailable. An investigation by the publisher verified that some references did not have available online records. Following additional investigation, all parties have concluded that this article was accepted solely on the basis of a compromised peer review process. In view of the clear evidence of compromised peer review, the parties agreed that the paper must be retracted. The authors did not respond to our notice regarding the retraction.

